# Loss-of-Function *GHSR* Variants Are Associated With Short Stature and Low IGF-I

**DOI:** 10.1210/clinem/dgaf010

**Published:** 2025-01-09

**Authors:** Lauren D Punt, Sander Kooijman, Noa J M Mutsters, Kaiming Yue, Daniëlle C M van der Kaay, Vera van Tellingen, Willie M Bakker-van Waarde, Annemiek M Boot, Erica L T van den Akker, Anneke A van Boekholt, Kirsten de Groote, Anne R Kruijsen, Nancy H G van Nieuwaal-van Maren, M Claire Woltering, Malou Heijligers, Josine C van der Heyden, Ellen M N Bannink, Tuula Rinne, Sabine E Hannema, Wouter J de Waal, Lucia C Delemarre, Patrick C N Rensen, Christiaan de Bruin, Hermine A van Duyvenvoorde, Jenny A Visser, Patric J D Delhanty, Monique Losekoot, Jan M Wit, Sjoerd D Joustra

**Affiliations:** Division of Pediatric Endocrinology, Department of Pediatrics, Willem-Alexander Children's Hospital, Leiden University Medical Centre, 2333 ZA Leiden, the Netherlands; Division of Endocrinology, Department of Medicine, Leiden University Medical Centre, 2333 ZA Leiden, the Netherlands; Einthoven Laboratory for Experimental Vascular Medicine, Leiden University Medical Centre, 2333 ZA Leiden, the Netherlands; Division of Endocrinology, Department of Internal Medicine, Erasmus MC, University Medical Centre Rotterdam, 3015 GD Rotterdam, the Netherlands; Division of Endocrinology, Department of Medicine, Leiden University Medical Centre, 2333 ZA Leiden, the Netherlands; Division of Pediatric Endocrinology, Department of Pediatrics, Erasmus University Medical Centre, Sophia Children's Hospital, 3015 GD Rotterdam, the Netherlands; Department of Pediatrics, Catharina Hospital, 5623 EJ Eindhoven, the Netherlands; Division of Pediatric Endocrinology, University Medical Centre Groningen, University of Groningen, 9713 GZ Groningen, the Netherlands; Division of Pediatric Endocrinology, University Medical Centre Groningen, University of Groningen, 9713 GZ Groningen, the Netherlands; Division of Pediatric Endocrinology, Department of Pediatrics, Erasmus University Medical Centre, Sophia Children's Hospital, 3015 GD Rotterdam, the Netherlands; Department of Pediatrics, VieCuri Medical Centre, 5912 BL Venlo, the Netherlands; Division of Pediatric Endocrinology, Department of Pediatrics, Willem-Alexander Children's Hospital, Leiden University Medical Centre, 2333 ZA Leiden, the Netherlands; Division of Pediatric Endocrinology, Department of Pediatrics, Willem-Alexander Children's Hospital, Leiden University Medical Centre, 2333 ZA Leiden, the Netherlands; Department of Pediatrics, Gelderse Vallei Hospital, 6716 RP Ede, the Netherlands; Department of Pediatrics, Reinier de Graaf Gasthuis, 2625 AD Delft, the Netherlands; Department of Clinical Genetics, Maastricht University Medical Centre, 6229 HX Maastricht, the Netherlands; Department of Pediatrics, Franciscus Gasthuis & Vlietland, 3045 PM Rotterdam, the Netherlands; Department of Pediatrics, Tergooi MC, 1212 VG Hilversum, the Netherlands; Department of Human Genetics, Radboud UMC, 6525 GA Nijmegen, the Netherlands; Department of Pediatric Endocrinology, Amsterdam UMC, location Vrije Universiteit, 1081 HV Amsterdam, the Netherlands; Amsterdam Gastroenterology Endocrinology Metabolism, 1081 HV Amsterdam, the Netherlands; Amsterdam Reproduction and Development, 1105 AZ Amsterdam, the Netherlands; Department of Pediatrics, Diakonessenhuis, 3582 KE Utrecht, the Netherlands; Department of Pediatrics, Amstelland Hospital, 1186 AM Amstelveen, the Netherlands; Division of Endocrinology, Department of Medicine, Leiden University Medical Centre, 2333 ZA Leiden, the Netherlands; Einthoven Laboratory for Experimental Vascular Medicine, Leiden University Medical Centre, 2333 ZA Leiden, the Netherlands; Division of Pediatric Endocrinology, Department of Pediatrics, Willem-Alexander Children's Hospital, Leiden University Medical Centre, 2333 ZA Leiden, the Netherlands; Department of Clinical Genetics, Leiden University Medical Centre, 2333 ZA Leiden, the Netherlands; Division of Endocrinology, Department of Internal Medicine, Erasmus MC, University Medical Centre Rotterdam, 3015 GD Rotterdam, the Netherlands; Division of Endocrinology, Department of Internal Medicine, Erasmus MC, University Medical Centre Rotterdam, 3015 GD Rotterdam, the Netherlands; Department of Clinical Genetics, Leiden University Medical Centre, 2333 ZA Leiden, the Netherlands; Division of Pediatric Endocrinology, Department of Pediatrics, Willem-Alexander Children's Hospital, Leiden University Medical Centre, 2333 ZA Leiden, the Netherlands; Division of Pediatric Endocrinology, Department of Pediatrics, Willem-Alexander Children's Hospital, Leiden University Medical Centre, 2333 ZA Leiden, the Netherlands

**Keywords:** GHSR, short stature, growth hormone, IGF-I

## Abstract

**Context:**

The growth hormone (GH) secretagogue receptor, encoded by *GHSR*, is expressed on somatotrophs of the pituitary gland. Stimulation with its ligand ghrelin, as well as its constitutive activity, enhances GH secretion. Studies in knockout mice suggest that heterozygous loss-of-function of *GHSR* is associated with decreased GH response to fasting, but patient observations in small case reports have been equivocal.

**Objective:**

This work aims to establish the phenotype of *GHSR* haploinsufficiency and its growth response to GH treatment.

**Methods:**

This case series includes 26 patients with short stature and heterozygous *GHSR* variants. Pathogenicity was studied in vitro using total protein levels, cell surface expression, and receptor activity in basal, stimulated, and inhibited states.

**Results:**

Ten different variants were identified, of which 6 were novel. Variants showed either partial or complete loss of function, primarily through loss of constitutive activity. Patients (aged 4.0-15.1 years) had proportionate short stature (height −2.8 ± 0.5 SDS), failure to thrive with low appetite (n = 4), a mean serum insulin-like growth factor-I (IGF-I) of −1.6 ± 0.7 SDS, and a normal stimulated GH response. Nine patients received GH treatment, showing a height gain of 0.9 ± 0.4 SDS after 1 year and 1.5 ± 0.4 SDS after 2 years (n = 5).

**Conclusion:**

This study combines phenotypical and functional data in a uniquely large group of children with short stature carrying *GHSR* variants, and shows their good response to GH treatment. The results strengthen the hypothesis of *GHSR*'s role in GH secretion.

Growth hormone (GH) is the major regulator of linear growth. Its pituitary production is influenced mainly by stimulation by GH-releasing hormone (GHRH) and inhibition via somatostatin. In 1996, another modifier of GH secretion was identified: ghrelin and its receptor GHSR (GH secretagogue receptor) (1). This G protein–coupled receptor is encoded by *GHSR*, which contains 2 exons (2), and is mainly expressed in the somatotroph cells of the pituitary gland and several hypothalamic nuclei (1, 3). It has 2 known isoforms, GHSR1a and GHSR1b, the latter having no known function. The natural ligand for GHSR is the acylated form of ghrelin, which is secreted from the submucosal cells of the stomach during fasting (2, 4, 5). Its antagonist is the liver-enriched antimicrobial peptide-2 (LEAP2) (6).

Ghrelin plays 2 major roles in the body. First, it has an orexigenic effect and is involved in reward-related behaviors, glucose homeostasis, and body weight control (7-9). Second, ghrelin causes an increase in GH secretion, through its direct action on GHSR in the pituitary somatotrophs, and indirectly by antagonizing somatostatin effects, inducing GHRH secretion, and repression of the inhibitory effect of insulin-like growth factor-I (IGF-I) on GH secretion (10). It ensures that even in the absence of the GHRH receptor, or during therapy with octreotide (a somatostatin analogue), rhythmic GH secretion can be observed (11-13), although it works best synergistically with GHRH (14, 15). Additionally, GHSR has a remarkably high constitutive (basal) activity, as it signals at 50% of its maximal capacity in the absence of ghrelin (16, 17).

In rodents lacking *Ghsr*, ghrelin fails to stimulate appetite and GH secretion, and they show lower serum IGF-I and GH levels, as well as lower body weight and length, although this varies between different knockout models (18-23). Mice carrying a heterozygous mutant *Ghsr* with intact ghrelin responsiveness show a milder phenotype, but still have decreased GH levels during fasting (22). Mice with the homozygous missense mutation p.(Ala203Glu), corresponding with p.(Ala204Glu) in humans, showed decreased GH levels, decreased femur and body length when aged only after 1 year, and sensitivity to fasting-induced hypoglycemia. As this variant has lower constitutive activity but intact receptivity to ghrelin, these results highlight the importance of the constitutive activity of GHSR (24).

The release of GH after administration of ghrelin was also demonstrated in humans, showing a much greater effect than with maximal dosages of GHRH (25-28) or other known stimulators of GH secretion including insulin, arginine, clonidine, levodopa, or glucagon (29, 30). Treatment with GHSR agonists additionally increased IGF-I and GH levels in healthy older individuals (31, 32), as well as in children and adults with GH deficiency (GHD) (33, 34). Subsequently, the GHSR agonist macimorelin was approved by the US Food and Drug Administration as a diagnostic agent for adult GHD (35).

Given the well-established importance of GHSR in GH release, variants in *GHSR* have been studied for their influence on height. Indeed, an association between *GHSR* variants and adult height has been shown in many (36-41), but not all (42-44), genome-wide association studies (GWAS). In a burden test within the UK Biobank (genebass.org), strong associations were identified between predicted loss-of-function variants in *GHSR* and both standing height (*P* = 1.9 × 10^−9^) and IGF-I levels (*P* = 3.9 × 10^−9^) (45). Although “isolated partial GHD” due to *GHSR* variants is a registered syndrome (MIM No. 615925), only a few cases have been published, showing a diverse clinical picture, incomplete cosegregation, and usually a lack of functional studies to assess pathogenicity of the variants (46). Subsequently, GHSR deficiency is not an approved indication for recombinant human GH (rhGH) therapy in many parts of the world, and there is a need for thorough characterization of the phenotype and evaluation of the effect of rhGH therapy in patients with true pathogenic variants. Therefore, we studied the pathogenicity of 10 variants in *GHSR* (6 novel) using in vitro analyses, and describe the phenotype of 25 patients carrying partial or complete loss-of-function variants and the response to rhGH therapy in 9 of them.

## Materials and Methods

### Design

The study is a descriptive case series. The medical ethics committee of the Leiden University Medical Centre issued a statement of no objection. Patients or their parents gave written consent for the collection and publication of data. Anonymized data are available on request.

### Participants

Patients with short stature carrying *GHSR* variants were collected from databases of laboratories for genome analysis in the Netherlands. Twenty-six patients from 24 families were included, harboring 10 different variants classified according to the American College of Medical Genetics and Genomics/Association for Medical Pathology criteria as variant of unknown significance or higher (47). Data were collected from patient records. Reference data were used to calculate birth weight and head circumference SD scores (SDS) (48), height SDS (49), target height SDS (50), predicted adult height (PAH) using Bonexpert V3.1 with appropriate ethnicity (51), sitting height/height index SDS (52), and body mass index (BMI) SDS (53). IGF-I SDS was calculated using a reported reference for children (54) and an in-house reference for adults. In all patients an extensive gene panel was performed to exclude other prevalent genetic causes of growth failure. A description of the molecular genetic analysis reference data is shown in the supplemental material (55). In 3 patients an IGF-I generation test was performed (56).

### Functional Studies

pCMV6-GHSR (Origene) was used for the generation of different plasmids encoding the GHSR variants necessary for subsequent assessment of transcriptional activity. In addition to N-terminally tag GHSR with HiBiT to assess cell surface expression, its complementary DNA (cDNA) was polymerase chain reaction–amplified from pcDNA3.1-GHSR using Phusion turbo polymerase (Thermo Fisher Scientific) and ligated into pBiT3.1-secN (Promega). For both assays *GHSR* variant sequences were generated by polymerase chain reaction site-directed mutagenesis using the QuikChange (Agilent) method, as previously described (57) and confirmed by Sanger sequencing.

To study the transcriptional activity associated with the different *GHSR* variants, HEK293 cells were cultured at 37 °C with 5% CO_2_ and transfected with 10-ng *GHSR* plasmid or a mock plasmid (pcDNA3.1) together with pSRE-Luc and pRenilla using FuGENE transfection reagent. The SRE reporter measures (Gαq driven) activity of the PLC/PKC-ERK pathway (58). These cotransfection experiments were performed in triplicates in 24-well plates at 80 000 cells/well, and replicated at least twice for each of the variants and the average result is presented. After 24 hours, cells were incubated for 6 hours in Dulbecco’s modified Eagle’s medium with or without the endogenous ligand ghrelin at the indicated concentrations, or in the presence of the inverse agonist (D-Arg1, D-Phe5, D-Trp7, 9, Leu11)-substance P analogue (SPA; 10^−6^ M). Luminescence was measured, and the data of each well were normalized to the Renilla value. Reported values are basal, stimulated and inhibited activity, corrected for the mock plasmid activity, and relative to wild-type (WT).

Along with these experiments, cell surface expression levels of GHSR at the plasma membrane were investigated using the HiBiT Extracellular Detection System (Promega). HEK293 cells were seeded in clear-bottom 96-well plates at a concentration of 20 000 cells/well. Cells were incubated for 24 hours at 37 °C with 5% CO_2_. After 24 hours, wells were transfected with 0.25 ng of HiBIT-GHSR plasmid and 50 ng of the transfection carrier DNA provided in the kit (Promega) using FuGENE. The next day, the medium was replaced with 50-µL CO_2_-free medium containing 0.5% fetal calf serum. To each well, 50 µL of LgBiT protein and HiBiT extracellular substrate (diluted 1:100 and 1:50, respectively, in HiBiT Extracellular buffer) was added. The plate was incubated for 10 minutes on a shaker, at 500 rpm. The luminescence was measured in a CLARIOstar Plus plate reader (BMG Labtech). Total levels of GHSR-HiBiT were assessed in the same way, but using the HiBiT Lytic Detection System (Promega). Experiments were performed as quadruplicates and replicated at least 3 times for each of the variants, and the average result is presented.

### Statistical Analysis

GraphPad Prism (version 10.2.3) was used to perform nonlinear curve fitting of the dose-response data, and for statistical tests. Comparisons between WT and variant were assessed using unpaired 2-tailed *t* tests, and a *P* value less than or equal to .05 was considered statistically significant.

## Results

We included 26 patients (19 boys and 7 girls) who were heterozygous for variants in *GHSR*, with an average age of 8.0 years (range, 4.0-15.1 years). One patient was excluded from the analysis of clinical and laboratory features because pathogenicity of the variant was uncertain after functional analyses. A summary of the characteristics of the remaining 25 patients is presented in Table 1, and individual clinical and biochemical characteristics of the patients are presented in Supplementary Table S1 (55).

**Table 1.** Summary of clinical and biochemical characteristics of patients

	No. of patients with available data	Average ± SDS or percentage
Total patients	25	
Male	18	72%
Female	7	28%
Preterm birth	21	14%
Birthweight SDS	22	−0.6 ± 1.2
Birth length SDS	16	−1.3 ± 1.4
At evaluation		
Age, y	25	8.0 ± 3.2
Height SDS	25	−2.8 ± 0.5
BMI SDS	25	−0.6 ± 1.0
HC SDS	22	−0.9 ± 1.0
SH/H SDS	24	0.7 ± 0.8
BA delay, y	21	1.2 ± 0.9
IGF-I SDS	25	−1.6 ± 0.7
IGFBP-3 SDS	15	−1.0 ± 0.8
GH peak*^a^*, µg/L	15	18.7 ± 6.5
ΔIGF-I SDS in IGFGT*^b^*	3	1.8 ± 0.9
Failure to thrive	25	16%
Developmental delay	25	8%
Mild dysmorphic features*^c^*	25	20%
Nonaffected parent height SDS	19	−1.0 ± 0.9
Affected parent height SDS	18	−1.0 ± 1.2
Target height SDS	25	−0.8 ± 0.7

Abbreviations: BA, bone age; GH, growth hormone; HC, head circumference; IGF-I, insulin-like growth factor-I; rhGH, recombinant human growth hormone; SDS, SD score; SH/H, sitting height divided by height.

^*a*^Maximum GH peak during arginine or clonidine stimulation test.

^*b*^IGF-I generation test: increment in IGF-I SDS after 7 days of 0.025 mg/kg.day of rhGH.

^*c*^See “Results” for description.

### *GHSR* Variants

**Figure 1.**
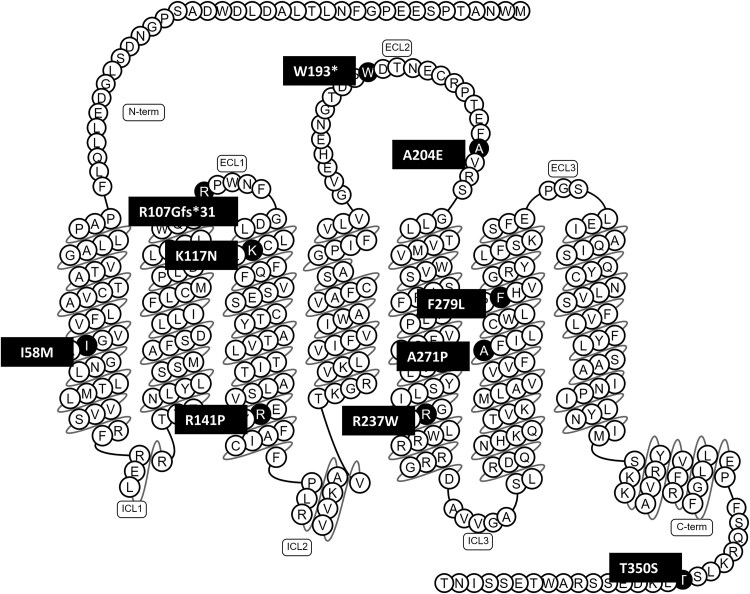
Snake-plot of variant locations within growth hormone secretagogue receptor (*GHSR*).

Eight missense variants (all variants of unknown significance), 1 frameshift, and 1 nonsense variant (both likely pathogenic) were identified in *GHSR* (Table 2). Fig. 1 shows that most variants are located in the transmembrane domains of the protein. Of the variants of unknown significance, all had very low frequency in population databases (p.(Arg237Trp) 0.01-0.02%, all others <0.01%). In silico prediction tools for missense variants supported a deleterious effect in 5 variants (p.(Lys117Asn), p.(Arg141Pro), p.(Arg237Trp), p.(Ala271Pro), and p.(Phe279Leu)), and equivocal predicted effects in p.(Thr350Ser) and p.(Ile58Met). Variant p.(Ala204Glu) was previously reported as pathogenic (59).

**Table 2.** Specification of (likely) pathogenic variants in *GHSR*

Patient No.	WT	26	1	2	3-8	9	10-21	22	23	24	25
cDNA change		c.174C > G	c.319del	c.351A > T	c.422G > C	c.578G > A	c.611C > A	c.709A > T	c.811G > C	c.837C > A	c.1049C > G
Protein change		p.(Ile58Met)	p.(Arg107Glyfs*31)	p.(Lys117Asn)	p.(Arg141Pro)	p.(Trp193*)	p.(Ala204Glu)	p.(Arg237Trp)	p.(Ala271Pro)	p.(Phe279Leu)	p.(Thr350Ser)
**In silico analyses**											
Type		Missense	Frameshift	Missense	Missense	Nonsense	Missense	Missense	Missense	Missense	Missense
ACMG criteria*^a^*		PM2 BP4	PM2 PVS1	PM2 PP3	PM2 PP3	PM2 PVS1	PM2 PP5	PM1 PM2 PP3	PM2 PP3	PM2 PP3	PM2 BP4 PP5
Classification		VUS	LP	VUS	VUS	LP	VUS	VUS	VUS	VUS	VUS
**In vitro analyses**											
Cell surface expression	100%	148%	30%*^g^*	60%*^f^*	10%*^g^*	3%*^g^*	54%*^f^*	198%	144%	92%	92%
Total protein levels	100%	170%	34%*^g^*	81%	22%*^g^*	45%*^f^*	37%*^f^*	144%	73%	69%*^f^*	69%*^f^*
Basal activity*^b^*	100%	115%	9%*^g^*	22%*^f^*	4%*^g^*	0%*^g^*	14%*^g^*	63%*^f^*	0%*^g^*	34%*^f^*	34%*^f^*
EC_50_*^c^*	2.9	1.6	<0.1	5.2	∞	1.5	9.3	1.9	29.8	3.9	3.9
Emax*^d^*	100%	91%*^f^*	4%*^g^*	128%*^f^*	4%*^g^*	0%*^g^*	161%*^g^*	85%*^f^*	88%	158%*^g^*	158%*^g^*
Imax*^e^*	100%	175%*^f^*	25%*^f^*	80%	32%*^f^*	0%*^g^*	77%*^f^*	152%*^f^*	28%*^f^*	195%*^f^*	195%*^f^*
**Summary**		Indefinite	Complete LoF	Partial LoF	Complete LoF	Complete LoF	Partial LoF	Partial LoF	Partial LoF	Partial LoF	Partial LoF

Abbreviations: ACMG, American College of Medical Genetics and Genomics; cDNA, complementary DNA; *GHSR*, growth hormone secretagogue receptor; LoF, loss of function; LP, likely pathogenic; VUS, variant of unknown significance; WT, wild-type.

^*a*^American College of Medical Genetics and Genomics classification (see Supplementary material (55)).

^*b*^Measured basal activity, as a percentage of the WT basal activity.

^*c*^Half-maximal effective concentration of synthetic ghrelin (nM).

^*d*^Maximum stimulation with synthetic ghrelin (10^−6^ M for all variants), as a percentage of the WT Emax.

^*e*^Maximum inhibition of GHSR with substance P (10^−6^ M for all variants), as a percentage of WT Imax.

^*f*^ *P* less than or equal to .05.

^*g*^ *P* less than or equal to .001.

Two variants of unknown significance were found in other genes, that is, a variant in *ACAN* in patient 1 and in *COMP* in patient 20. See the supplemental material for more information on these variants (55).

### Functional Analysis of Variants

**Figure 2.**
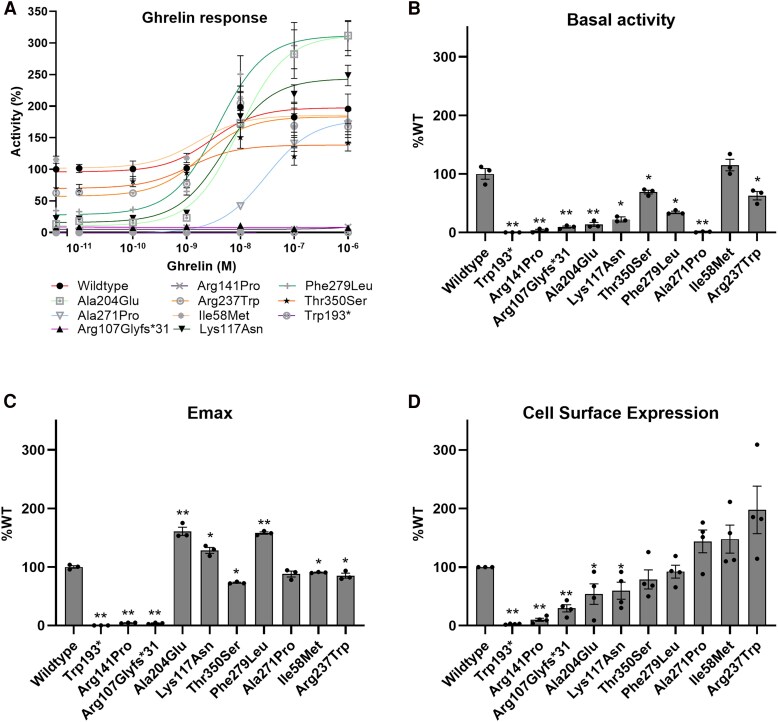
Functional assessment of growth hormone secretagogue receptor (*GHSR*) variants. A, *GHSR* variant responses to ghrelin dose curves, with baseline level expressed relative to wild-type baseline. B, Basal activity. C, Emax (stimulated activity). D, Cell surface expression. Data are expressed as percentage wild-type ± SEM of 4 to 6 independent experiments. *P less than or equal to .05; **P less than or equal to .0001.

Maximum stimulation of the WT receptor with ghrelin increased its activity to 191% (Fig. 2A) while inhibition with substance P led to a decrease in the activity to 61%. Variants p.(Arg141Pro), p.(Arg107Glyfs*31), and p.(Trp193*) showed a complete loss of cell surface expression (ie, ≤ 30% of WT), basal activity (ie, ≤ 30%), and stimulated activity (*ie,* Emax ≤30%) and were considered complete loss-of-function variants (see Table 2 and Fig. 2B-2D).

Missense variants p.(Lys117Asn), p.(Ala204Glu), p.(Arg237Trp), p.(Ala271Pro), p.(Phe279Leu), and p.(Thr350Ser) showed complete or partial loss in basal activity (ie, ≤ 70%) with either normal or partial loss (ie, ≤ 70%) of cell surface expression. In addition, p.(Arg237Trp) and p.(Thr350Ser) showed a slight decrease in stimulated activity. For the others, the response to ghrelin was unimpaired, although EC_50_ was shifted to the right in (Lys117Asn), p.(Ala204Glu), p.(Phe279Leu), and p.(Ala271Pro). All 6 were considered partial loss-of-function variants.

The p.(Ile58Met) variant of patient 26 resulted in slightly lower stimulated activity (91%; *P* < .001). Cell surface expression was increased at 148%, though not statistically significant (*P* = .093). One might expect a corresponding high-normal basal activity in the transfected cells, but this was not the case (ie, 115%). Given the equivocal results, the effects of the variant on receptor functioning are uncertain. Clinical characteristics of this patient are presented in the supplemental material (55).

### Growth and Puberty

**Figure 3.**
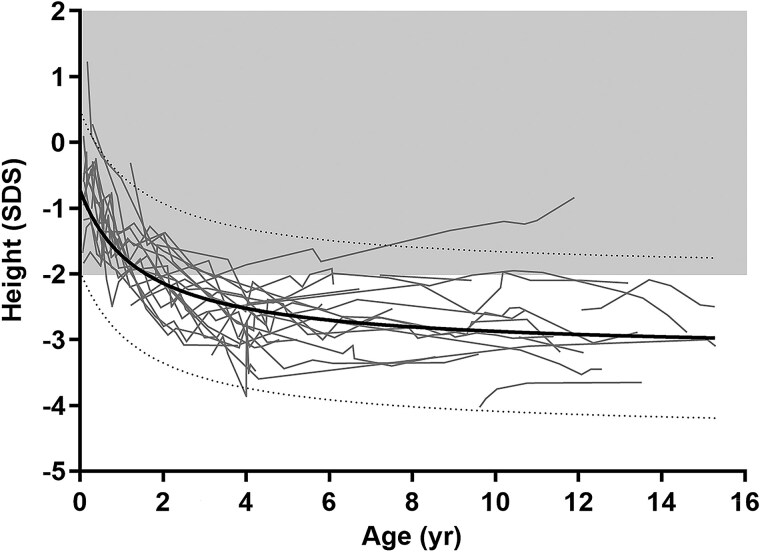
Height SDS in patients not on recombinant human growth hormone therapy. The black curve represents the fitted average, and the dotted curves represent the 95% prediction interval.

Patients generally had a normal birth length with decreasing height SDS during the first 2 years of life (Fig. 3). Average height at presentation (age range, 4.0-15.1 years) was −2.8 SDS (range, −4.1 to −1.9 SDS). An increased sitting height/height index SDS was present in patient 25.

**Figure 4.**
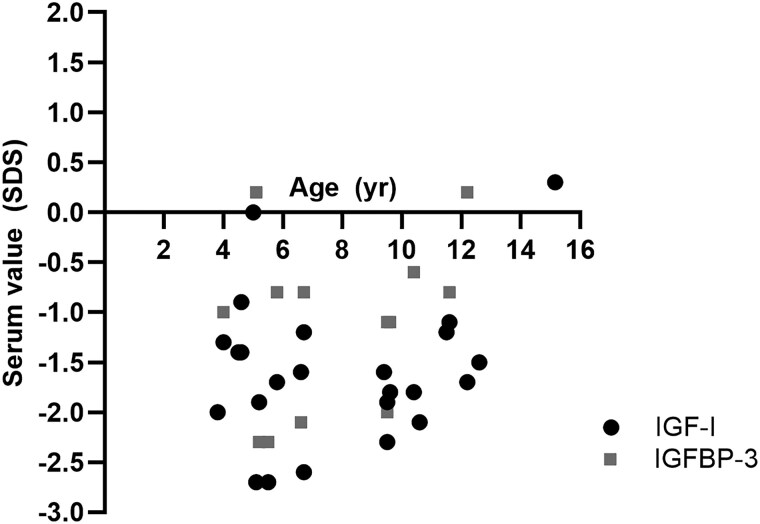
Serum insulin-like growth factor-I (IGF-I) and IGFBP-3 SDS of each patient at presentation, before start of recombinant human growth hormone therapy.

The average serum IGF-I and IGFBP-3 were −1.6 SDS (range, −2.7 to 0.3 SDS) and −1.0 SDS (range, −2.3 to 0.2 SDS), respectively (Fig. 4). A GH stimulation test was performed in 15 patients and all showed a sufficient or even exaggerated rise (range, 10.8-32.7 µg/L). An IGF-I generation test in 3 patients showed an IGF-I rise of +1.2, +1.3 SDS, and +2.8 SDS after 7 days of 0.025 mg/kg.day of rhGH.

Patients 6, 13, and 20 were born prematurely, with maternal HELLP syndrome (hemolysis, elevated liver enzymes, and low platelet count occurring in association with preeclampsia) in patient 13 as a contributing factor. Five were born small for gestational age (SGA). Contributing maternal factors were a very small placenta in patients 11 and 8 and tobacco use in patient 1. Pubertal development was generally unremarkable. Twelve patients started puberty at a normal age: 7 boys at 10.0 to 13.5 years and 5 girls at 11.0 to 12.4 years. Eleven patients were still prepubertal at latest evaluation, the oldest girl being age 11.1 years and the oldest boy 11.0 years. Patient 11 had early thelarche at age 7.5 years and patient 7 late gonadarche at age 14.6 years.

### Other Phenotypic Features

Average BMI at presentation was normal (−0.6 SDS, range −2.6 to 2.1 SDS), with slightly lower BMI in early life (BMI at age 2 years, n = 12: −1.1 ± 1.4 SDS). Patients 1, 4, 6, and 7 had failure to thrive with low appetite, for which the latter 2 received nasogastric tube feeding for a short period.

Developmental delay was reported in 2 patients. Patient 11 had an IQ of 55 and attends special primary education, as does patient 8, who had an IQ of 57 and attention deficit hyperactivity disorder. No explanation for the developmental delay was found with extensive genetic evaluation, including array and whole-exome sequencing.

In addition, the treating physician reported clinodactyly of the third finger and a sandal gap of both feet in patient 5, a slightly long philtrum in patient 11, and mild frontal bossing and hypermobility in hands and feet in patient 12. Patient 1 had an epicanthic fold, small almond-shaped eyes, a thin upper lip, bilateral mild conductive hearing loss, dyslexia, and microcephaly (−2.5 SDS). She had prenatal alcohol exposure, but insufficient criteria for fetal alcohol syndrome were met. Patient 9 was born with a distal reduction defect of his right arm, patient 3 suffered a femur fracture at birth, patient 10 was diagnosed with myotonic dystrophy, patient 6 with Hashimoto disease at age 8 years and was treated adequately with levothyroxine. Patient 17 was diagnosed with celiac disease at age 15 years. He had short stature since kindergarten, normal BMI, and no gastrointestinal complaints. Patient 20 was born with perineal hypospadias and unilateral cryptorchidism; extensive genetic screening for disorders of sexual development gave no explanation. He furthermore was diagnosed with attention deficit hyperactivity disorder at age 8 years.

### Genotype-Phenotype Correlation

In 17 of 23 families, genetic evaluation was available for both parents. In 7 families the variant segregated with short stature, as the affected parent had a height between −1.5 and −2.0 SDS (n = 3) or < –2.0 SDS (n = 4). In one of them (patient 13), the affected parent had been treated with rhGH from ages 5 to 15 years within a study context because of familial very short stature. In the remaining 10 families, short stature was absent in parents either with or without the variant (height −1.4 to 1.1 SDS and −1.5 to 1.0 SDS, respectively). In 2 families, multiple siblings had short stature and carried the p.(Ala204Glu) variant. In one of these families there was no short stature in either the parent with or without the variant, and in the other family no genetic evaluation of the parents was performed. Based on anecdotal reports, a growth pattern resembling constitutional delay of growth and puberty may have been present in the father of patient 3 (height −3.7 SDS at age 16 years), patient 5, and patient 7 (as well as the patient's brother), all carrying the p.(Arg141Pro) variant.

### Response to Recombinant Human Growth Hormone Treatment

**Figure 5.**
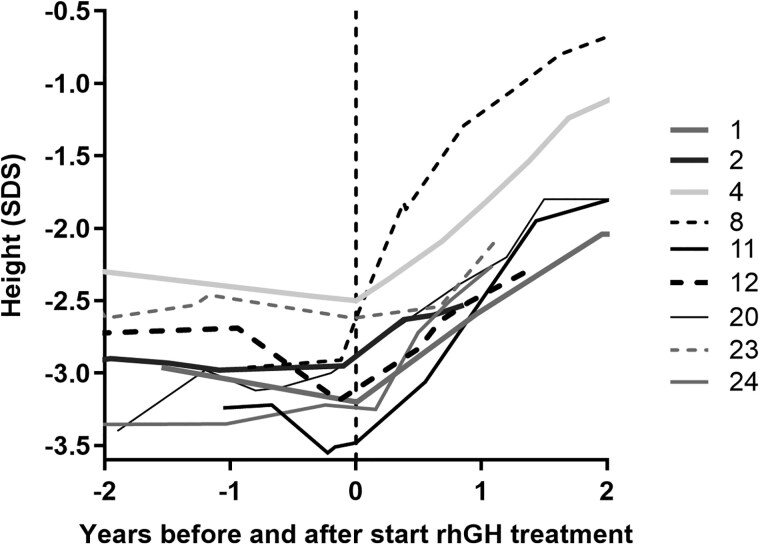
Height SDS during the 2 years before and after start of recombinant human growth hormone (rhGH) therapy

Nine patients had received rhGH therapy (Table 3). Seven individiuals were prepubertal when they started therapy. After 1 year, patient 24 had a testicular volume of 6 to 8 mL, patient 12 had thelarche, and patient 2 had a testicular volume of 4 mL. Their average gain in height after 1 year was 0.9 SDS (range, 0.5-1.6 SDS; Fig. 5), the gain in PAH was 1.0 SDS (range, 0.6-2.2 SDS), and the increase in height velocity was +4.6 cm/year (range, +3.5-6.6 cm/year). Patient 4 (male) was at Tanner stage G3 to G4 at the start and was treated with an aromatase inhibitor before and during rhGH therapy, and patient 1 (female) was at Tanner stage B3 at the start. After 1 year, their height improved by respectively +0.7 and +0.6 SDS, their PAH by +2.2 and +0.6 SDS, and their annualized height velocity (HV) by +4.6 and +4.9 cm/year.

**Table 3.** Response to recombinant human growth hormone treatment in patients with (likely) pathogenic variants in *GHSR*

	1	2*^a^*	4*^b^*	8	11*^c^*	12*^d^*	20	23	24*^e^*	Total
Variant	p.(Arg107Glyfs*31)	p.(Lys117Asn)	p.(Arg141Pro)		p.(Ala204Glu)			p.(Ala271Pro)	p.(Phe279Leu)	
**At start of rhGH therapy**										
Age, y	12.4	11.5	15.2	4.6	4.6	11.7	4.9	7.0	9.9	9.1 ± 3.9
Sex	Female	Male	Male	Male	Female	Female	Male	Male	Male	
Height (SDS)	−3.2	−2.9	−2.5	−2.9	−3.4	−3.2	−3.0	−2.6	−3.3	−3.0 ± 0.3
HV, cm/y	4.4	4.0	4.5	8.1	5.3	4.0	6	4.6	4.6	5.1 ± 1.3
PAH, SDS	−2.9	−3.3	−2.4	—	−3.8	−2.2	—	—	−3.3	−3.0 ± 0.6
BA delay, y	1.4	0.0	0.5	—	0.6	1.8	—	—	1.2	0.9 ± 0.7
Tanner stage	3	1	3-4	1	1	1	1	1	1	
IGF-I, SDS	−2.1	−0.7	−1.7	−1.4	−2.0	−1.1	0.0	−1.7	−1.0	−1.3 ± 0.7
Starting dose, mg/kg.d	—	0.037	0.032	0.043	—	0.028	—	0.028	0.035	0.034 ± 0.006
**After 1 y of rhGH**										
Height gain, SDS	0.6	0.4	0.7	1.6	1.5	0.8	0.7	0.5	1.1	0.9 ± 0.4
PAH gain, SDS	0.6	0.7	2.2	—	—	0.6	—	—	0.9	1.0 ± 0.7
HV 0-1 y, cm/y	9.3	7.8	9.1	12.1	11.8	9.3	8.8	8.1	11.2	9.7 ± 1.6
BA delay, y	1.0	0.4	3.1	—	—	1.8	—	—	0.2	1.3 ± 1.2
Dose after 1 y, mg/kg.d	—	0.035	0.020	0.036	—	0.026	—	0.053	0.032	0.034 ± 0.011
**After 2 y of rhGH**										
Height gain SDS 0-2 y	1.1		1.4	2.1	1.6		1.2			1.5 ± 0.4
PAH gain SDS 0-2 y	1.6		2.4	—	—		—			2.0 ± 0.6
HV 1-2 y, cm/y	6.4		6.9	8.2	7.1		9.9			7.7 ± 1.4
BA delay, y	2.0		3.5	1.1	—		—			2.2 ± 1.2
Dose after 2 y, mg/kg.d	0.036		0.012	0.037	—		—			0.028 ± 0.014
**At stop of rhGH therapy**										
Age, y			18.8		14.53					
Height, SDS*^f^*			−0.6		−1.7					
BA delay, y			—		—					
Tanner stage			5		5					

Abbreviations: BA, bone age; GH, growth hormone; GnRH, gonadotropin-releasing hormone; HV, annualized height velocity; IGF-I, insulin-like growth factor-I; PAH, predicted adult height; SDS, SD scores.

^*a*^Testicular volume 6 to 8 mL at latest visit, 1 year after start of GH.

^*b*^Aromatase inhibitor from ages 13.0 to 17.8 years.

^*c*^Thelarche at 7.5 years, GnRH analogue from 7.5 to 12.3 years.

^*d*^Thelarche at 12.4 years, started GnRH analogue.

^*e*^Gonadarche at 12.2 years.

^*f*^SDS for adult height (reference age, 21 years).

During the second year, patients 11, 8, and 20 were still prepubertal and showed an increase in height of +1.2-2.1 SDS during the 2 years of treatment. Patients 4 and 1 were pubertal during the second year of treatment, and showed a total increase in PAH of +2.4 and +1.6 SDS in 2 years.

Patients 4 and 11 were treated until near-adult height. Patient 11 was cotreated with a gonadotropin-releasing hormone (GnRH) agonist from age 7.5 to 12.3 years. At treatment discontinuation, height SDS (for adult height, reference age 21 years) of patients 4 and 11 was −0.6 and −1.7 SDS, respectively, which was 1.9 and 1.7 SDS taller than height SDS at start, and 1.8 and 2.1 SDS taller than PAH at start.

## Discussion

We studied 10 different variants in *GHSR* in 26 patients from 24 families. Six of these variants were novel. The p.(Arg141Pro), p.(Arg107Glyfs*41), and p.(Trp193*) variants showed complete loss of function and the p.(Lys117Asn), p.(Ala204Glu), p.(Arg237Trp), p.(Ala271Pro), p.(Phe279Leu), and p.(Thr350Ser) variants partial loss of function. Genotypes showed haploinsufficiency with incomplete penetrance, segregating with short stature, or previous use of GH in 5 of 17 families with available data. The reason for the incomplete penetrance is unknown. Extensive gene panels and biochemical analyses were used to exclude other prevalent causes of growth failure in the studied patients. We hypothesize that variants in *GHSR* significantly reduce height, but that this happens within the background of the huge natural variation of height. Recent GWAS data in 5.4 million people show that single-nucleotide variations in 7209 different regions of the genome, covering 21% of the entire human genome, affect human height to some extent (36). This may result in a variable phenotype with absence of short stature in some carriers. In addition, assortative mating may contribute to a greater height reduction with every generation.

Given the in vitro loss of function, the in silico prediction of these variants now includes American College of Medical Genetics and Genomics criterion PS3 (47), all variants of unknown significance are reclassified as likely pathogenic, and the 2 likely pathogenic variants as pathogenic. The p.(Ile58Met) variant showed equivocal effects on receptor functioning both in the in silico and in vitro analyses, and this patient was therefore excluded from the analysis of clinical features. However, this variant showed a small but statistically significant decrease in stimulated activity, which over time might cause clinically relevant growth failure. The patient responded well to rhGH therapy (+1.4 SDS in height after 2 years). Therefore, this variant does warrant further functional studies before its pathogenicity is definitely rejected or accepted.

Patients generally showed proportionate short stature (−2.8 ± 0.5 SDS), low serum IGF-I (−1.6 ± 0.7 SDS) with considerable variability, a normal stimulated GH response (performed in 15), and 4 patients suffered from failure to thrive with low appetite in infancy. Our results are in accordance with previous case reports, as summarized in Supplementary Table S2 (55). The p.(Ala204Glu) variant was previously reported in a child with obesity (60), with no information on height or the age of onset of obesity. Next, it was reported in a family with short stature, low IGF-1, and GHD in some, including one homozygous patient with extremely short stature (59). The p.(Ala204Glu) variant was reported in 4 families with GHD (61) and was previously shown to have an 80% decrease in cell-surface expression with impaired constitutive activity but preserved responsiveness to ghrelin (37, 59). We did not observe GHD, but a pattern of constitutional delay of growth and puberty may have been present in the affected fathers of patients 3, 5, and 7, who all carry the p.(Arg141Pro) variant. Ten patients did not undergo a GH stimulation test, but there were no differences in growth response to rhGH between patients with (n = 3) or without a previous GH stimulation test (n = 6). The p.(Phe279Leu) variant was reported in a child with short stature (60) and showed lower constitutive activity (37). The p.(Arg237Trp) variant was previously described in a patient with compound heterozygosity for this variant and p.(Trp2X), showing short stature, low BMI, and very low IGF-I (62). The p.(Arg237Trp) variant was also present in his brother, who had an IGF-I of −1.0 SDS and was 1.5 SDS shorter than an unaffected third brother. The variant showed decreased constitutive activity (62). Last, the p.(Thr350Ser) variant was previously reported as likely pathogenic in a patient with short stature (63), and recently reported by our group in a patient with short stature, low IGF-I, and IGFBP-3 but normal stimulated GH, a very good effect of GH therapy, and good segregation with short stature (64). The other 6 variants in our manuscript have not been reported before.

Nine patients in our study received rhGH treatment, resulting in an average increase of 0.9 ± 0.4 SDS in height after 1 year, an increase of 1.5 ± 0.4 SDS after 2 years (n = 6), and an increase of 1.7 to 1.9 SDS on reaching adult height (n = 2). Only a few reports have described the effect of rhGH treatment. Two papers reported rhGH therapy in patients with the p.(Ala204Glu) variant and observed a good response without further specification (59, 61). A 6-year-old boy with compound heterozygosity for 2 variants (1 truncating and 1 missense variant) showed an increase of +1.7 SDS in height after 2 years of treatment (62). We recently reported a height increase of +0.7 SDS after 9 months of treatment in a boy with the p.(Thr350Ser) variant (64).

Our observations of proportionate short stature, low IGF-1, and good response to rhGH are in line with the reported function of GHSR: increasing GH secretion both in its constitutive state and after stimulation with ghrelin, and fit with the hypothesis of GH neurosecretory dysfunction in patients carrying pathogenic variants. GH stimulation tests generally yield normal results, as the traditionally used pharmacological agents act by stimulating GHRH or inhibiting somatostatin, pathways that are intact in these patients. Our observation of developmental delay in 2 patients has not been reported before, and it remains unclear if this is related to the *GSHR* variants; no alternative cause was identified despite extensive genetic evaluation although this is common in developmental delay (65). We observed failure to thrive with low appetite in 4 patients, 3 of whom had the p.(Arg141Pro) variant and 1 who the truncating variant. These variants were among the three with the lowest cell surface expression, and therefore showed both impaired constitutive activity and ghrelin responsiveness.

One might wonder why hunger and GH secretion would be physiologically related. It has been hypothesized that GH plays a pivotal role in glucose homeostasis during prolonged fasting, as it stimulates gluconeogenesis and glycogenolysis, triggers hydrolysis of triglycerides in adipose tissue with the release of free fatty acids, and antagonizes insulin (66, 67). No signs of hypoglycemia were reported in our patients. Evidence for a feedback loop between GH secretion and ghrelin is presented by the observation of increased ghrelin levels in GHD and GH insensitivity (68), and the decrease in ghrelin levels after the start of rhGH therapy (69, 70). Unfortunately, in this retrospective study, ghrelin levels were not available.

A major strength of our study is that the relatively large group of individuals with this rare disease doubles the number of previously reported patients and includes 6 novel variants with decreased receptor functioning. The inclusion of an in vitro assessment of pathogenicity strengthens our hypothesis that the observed phenotype is caused by the patient's *GHSR* variant. Moreover, the detailed description of the effect of rhGH therapy in 10 patients underlines the treatment potential. A possible limitation of the study is that the proposed relationship between short stature and *GHSR* variants may be subject to selection bias, as only patients with short stature have been subject to genetic evaluation. However, GWAS studies, observations in mice with suppressed GHSR function, studies of GH secretion after GHSR agonists in humans, and our segregation analysis (although with variable penetrance) all support this relationship. Also, our in vitro results support decreased functionality, though our model cannot assess GH release. Finally, in 2 patients an additional variant of unknown significance in either *ACAN* or *COMP* was found. However, especially the *ACAN* variant showed benign in silico characteristics, and neither patient showed a phenotype of family segregation consistent with pathogenicity. We consider it unlikely that these variants contributed to each patient's phenotype.

The results of this study are important because patients with pathogenic *GHSR* variants usually show short stature without a SGA-type growth pattern and an adequate stimulated GH peak. Therefore, without an established relationship between *GHSR* variants and treatable short stature, these patients would not be eligible for rhGH therapy in most parts of the world. Also, the results exemplify the importance of GHSR in GH secretion, and therefore provide additional support for investigating treatment with oral GHSR agonists in patients with a relatively intact pituitary axis, for example, those born SGA (71). These compounds, such as macimorelin, may also be used to assess the pathogenicity of *GHSR* variants. For instance, one patient homozygous for a p.(Leu90Pro) variant in *GHSR*, with severe short stature and an IGF-I of −2.8 SDS, showed no GH response to macimorelin, and her heterozygous siblings showed a blunted response (72). However, macimorelin may only test the effect of stimulation of the receptor and not assess its constitutive activity.

In conclusion, we studied 26 patients with 10 variants in *GHSR*. Nine variants showed in vitro decreased constitutive activity and/or decreased cell surface expression. Patients with partial or complete loss-of-function variants (n = 25) exhibited short stature, low IGF-I with a normal stimulated GH peak, and failure to thrive with decreased appetite in 15%. Treatment with rhGH therapy significantly increased linear growth. The results strengthen the hypothesis that pathogenic *GHSR* variants cause GH neurosecretory dysfunction, a treatable cause of short stature.

## Data Availability

Anonymized data are available on request.

## Abbreviations

BMI: body mass index
cDNA: complementary DNA
GH: growth hormone
GHD: growth hormone deficiency
GHRH: growth hormone–releasing hormone
*GHSR*: growth hormone secretagogue receptor
GnRH: gonadotropin-releasing hormone
GWAS: genome-wide association studies
HV: annualized height velocity
IGF-I: insulin-like growth factor-I
PAH: predicted adult height
rhGH: recombinant human growth hormone
SDS: SD scores
SGA: small for gestational age
