# Correction to: “Loss-of-Function GHSR Variants Are Associated With Short Stature and Low IGF-I”

**DOI:** 10.1210/clinem/dgaf083

**Published:** 2025-02-11

In the above-mentioned article by Punt LD, Kooijman S, Mutsters NJM, Yue K, van der Kaay DCM, van Tellingen V, Bakker-van Waarde WM, Boot AM, van den Akker ELT, van Boekholt AA, de Groote K, Kruijsen AR, van Nieuwaal-van Maren NHG, Woltering MC, Heijligers M, van der Heyden JC, Bannink EMN, Rinne T, Hannema SE, de Waal WJ, Delemarre LC, Rensen PCN, de Bruin C, van Duyvenvoorde HA, Visser JA, Delhanty PJD, Losekoot M, Wit JM, and Joustra SD (*J Clin Endo Metab*. 2025; doi: 10.1210/clinem/dgaf010), there was an error in the References section.

In the originally published article, in the References section, reference 55 read “Supplemental material to be uploaded to repository.” The authors have provided the URL for the supplemental material for their article, and it has been added to the reference citation. Reference 55 now reads “Supplemental material to be uploaded to repository. https://osf.io/nj6vu/.”

The article has been corrected online.

doi: 10.1210/clinem/dgaf010

